# Decolonization in Sexual and Reproductive Health Research Methods: Protocol for a Scoping Review

**DOI:** 10.2196/45771

**Published:** 2023-04-14

**Authors:** Maya Stevens-Uninsky, Aisha Barkhad, Tonya MacDonald, Alexander Perez, Lawrence Mbuagbaw

**Affiliations:** 1 Department of Global Health McMaster University Hamilton, ON Canada; 2 Department of Health Research Methods, Evidence and Impact McMaster University Hamilton, ON Canada; 3 Department of Anesthesia McMaster University Hamilton, ON Canada; 4 Department of Pediatrics McMaster University Hamilton, ON Canada; 5 Biostatistics Unit Father Sean O’Sullivan Research Centre St Joseph’s Healthcare Hamilton, ON Canada; 6 Centre for the Development of Best Practices in Health Yaoundé Central Hospital Yaoundé Cameroon; 7 Division of Epidemiology and Biostatistics Department of Global Health Stellenbosch University Stellenbosch South Africa

**Keywords:** sexual and reproductive health, decolonized research, decolonized methodologies, community-centered research, indigenous research, scoping review, electronic database, colonialism, decolonization, indigenous peoples, decolonizing methodologies, decolonizing method, human sexuality, reproduction, reproductive health, sexual and reproductive health, Aboriginal

## Abstract

**Background:**

As researchers and implementors begin to acknowledge the repercussions of institutionalized colonialism on community and individual health, the need to decolonize research has become clear. Despite this, there is neither a singular definition of decolonizing methodologies nor an overview of the shared principles and characteristics of decolonized research needed to codify this work as common practice in global health.

**Objective:**

The review will identify papers that reference principles of decolonization and identify shared characteristics between them. The aim of this scoping review is to review decolonized research methodologies through the lens of sexual health as a step in creating a shared understanding of best practices. We will further examine the tools and methods used to collect and analyze data within the included studies.

**Methods:**

The protocol for this scoping review was developed using the framework from the Joanna Briggs Institute and the PRISMA-ScR (Preferred Reporting Items for Systematic Reviews and Meta-Analyses Extension for Scoping Reviews). The search strategy will comprise a search of electronic databases (JSTOR, Embase, EMCare, MEDLINE [Ovid], Global Health Database, Web of Science), gray literature sources, and key studies. Titles and abstracts will be reviewed by 2 or more independent reviewers against inclusion criteria. Bibliometric details, study design, methodology, community involvement, and other indicators will be collected using a data extraction tool developed for this review. Extracted data will be analyzed using descriptive statistics and qualitative analysis of content and themes to identify common practices in decolonized methodologies within sexual health. Narrative summaries will be used to describe results in relation to the research question, and identified gaps will be discussed.

**Results:**

The initial title or abstract review of 4967 studies identified by the search strategy was completed in November 2022. In total, 1777 studies met initial inclusion criteria and were sent to a second round of title or abstract review, which was completed in January 2023. In total, 706 studies were downloaded for full-text inclusion, which is expected to be completed by April 2023. We aim to complete data extraction and analysis by May 2023 and expect to publish the findings by the end of July 2023.

**Conclusions:**

There remains a gap in the research on the meaning and application of decolonized research strategies, particularly within sexual and reproductive health. The findings of this study will contribute to a shared definition of decolonized methodologies and how they can be applied as a common practice in global health research. Applications include the development of decolonized frameworks, theoretical discourses, and methodologies. The study will inform the design and implementation of future decolonized research and evaluation strategies, particularly around sexual and reproductive health.

**International Registered Report Identifier (IRRID):**

DERR1-10.2196/45771

## Introduction

### Background

As researchers and implementors in the global health and development sector begin to acknowledge the repercussions of institutionalized colonialism, the call for the decolonization of research has grown [1]. Indigenous peoples globally have felt the othering effects of traditional research and implementation strategies. These strategies often frame them as numbers in a study designed by those operating within a dominant culture rather than individuals with unique perspectives and experiences. The colonialization of research manifests itself through the identification of funding priorities and recipients, academic imperialism, methodological imperialism, dominant culture definitions of success, and not least through the prominent use of western research ideals in research design and implementation. Funding for global health originates primarily in high-income countries where it shapes research priorities and establishes the measures and indicators of success without the involvement of communities of focus [2]. Western research paradigms, such as positivism or constructivism, as well as individualist roots, have shaped the way research and knowledge are conducted and perceived, superseding culturally relevant indigenous worldviews, learning, and language [3].

This approach to research serves to undermine indigenous knowledge and extracts data from communities without the intent of directly benefitting them [1], often removing them from accessing and using the information gathered. Relying exclusively on the western paradigm may also result in the use of evaluation and research tools that do not collect accurate and valid responses [4]. A unified definition of decolonizing methodologies and an overview of their shared principles and characteristics (or lack thereof) are necessary to codify this work as a common practice in global health research. This review will examine the meaning of decolonizing research by reviewing research methods, designs, and evaluation strategies through the lens of sexual health to identify the strategies used to implement and measure decolonized research.

### Decolonization of Research

The premise of decolonization is one of critiquing existing power structures and dominant culture at multiple levels. The approach is not centered specifically on human rights, sexual autonomy, or social justice. Instead, decolonization is a method of centering research, methodology, and practice within indigenous communities rather than rooting research in colonized institutions and epistemologies [5]. This scoping review defines decolonizing research as the prioritization of the needs and voices of indigenous communities in research, methodology, and practice in order to improve the caliber of assessment, analysis, and evaluation [6]. As authors such as Battiste [7] and Smith [5] emphasize, to decolonize research, it is of the utmost importance that indigenous communities and their ways of knowing lead research and that their voices are central to the process. Research paradigms should align with localized and indigenous ways of knowing to reclaim research and knowledge for indigenous peoples [3]. The decolonization of research methods will lead to indigenous research methods and provide a critique of dominant culture methodologies [5]. Decolonized research does not inherently mean oppositional to western methods but rather learning equally from dominant culture and indigenous methods in order to identify what is most applicable to a specific community.

While the need to decolonize research and practice has been identified and entered into the academic discourse, there is neither a clear definition for what a decolonized research methodology should entail or what elements these methodologies share nor an existing strategy to enter decolonized research methods into practice or best practice recommendations [8]. In particular, there is a need to identify these commonalities so that research strategies may be developed alongside communities to fit their specific needs, based on a body of foundational learning around decolonizing methodologies [9].

Research that is not rooted in decolonization can, and often will, result in the continued oppression of indigenous communities. These practices result in researchers and implementors entering communities and taking what they want, leaving behind little to no positive change and othering that community as a source of information rather than centering them in the work [10].

### Defining Indigenous

This research frequently uses the term indigenous in reference to the communities and populations who would initially benefit from the process of decolonizing research. While there is no universally agreed-upon definition of indigenous, the United Nations proposes “those which, having a historical continuity with pre-invasion and pre-colonial societies that developed on their territories, consider themselves distinct from other sectors of the societies now prevailing on those territories, or parts of them” [11]. Based on this commonly used definition where indigenous peoples are those who existed precolonial intervention, this research uses the terminology indigenous to refer to all of the first peoples of colonized nations.

The term indigenous is also often used to refer to a minority population or a population whose practices and social norms differ from the dominant culture. In the context of decolonization, it is important to acknowledge that colonialism has had lasting repercussions on the educational systems, research institutions, and governing bodies of the majority of the world. The argument can easily be made that the dominant culture and the institutions which uphold it do not reflect the cultures of even majority indigenous peoples. Colonialism has also had a lasting impact not only on indigenous peoples but also on formerly enslaved peoples who continue to live in spaces where colonial history has systemic ramifications on their health and well-being.

This review wishes to acknowledge the importance of indigenous research and knowledge within the context of decolonized research while still making an important distinction between the two. For the purpose of this work, indigenous research methods provide location or community-specific strategies and relationships to knowledge and data collection. Decolonization of research itself is the process of acknowledging and subsequently counteracting historical systems of power within and through the research being conducted. The two are linked, and in many scenarios, inextricable, but by no means the same.

### Sexual and Reproductive Health

The recognition of colonial history, the need to decolonize research methods, and the importance of centering the voices of the community in research are of particular importance when it comes to sexual and reproductive health (SRH). Colonialism and its institutions have historically oppressed women and sexual minorities, removing the capacity for choice and agency in family planning. Colonial powers have a history of the oversexualization of indigenous women [12], gynecological experimentation, eugenics, forced sterilization [13], population control [14], homophobia [15], and more, all of which are echoed in an ongoing culture of medical experimentation in previously colonized nations [16]. This history of oppression has ramifications today in the form of perceptions around access to SRH services, reliability of institutions, discrimination in service delivery, and desire to access SRH services. As such, reproductive justice and freedom of choice are critical areas of focus for decolonization research [17].

SRH rights and services are a broad range of fundamental health rights that apply to the physical and mental health of all individuals. SRH applies to a variety of topics that include access to family planning services, sexual health information, autonomy of sexual health decision-making, pregnancy and prenatal care, access to a trained health physician throughout pregnancy and birth, prevention and treatment of sexually transmitted infections, prevention of gender-based violence, abortion services [18], the ability to freely express sexual and gender identities [19], menstrual health education and access to products, and community information on menstruation and other sexual health subjects [20]. Lack of access to SRH services can result in poor mental and physical health outcomes.

SRH is divided by sex and gender and riddled with challenging power dynamics. Gender inequality and norms affect the ability of certain populations, particularly those who identify as women or gender nonconforming, to access equitable service delivery [21]. Specific SRH outcomes, such as teenage pregnancy, unintended or complex pregnancies, underage marriage, or domestic violence, predominantly affect those who identify as women or have uteruses [22].

Colonialism is a key determinant of health outcomes and therefore must be explicitly acknowledged in research methodologies [23]. To conduct relevant and effective research on sexual health in previously colonized nations, the needs of indigenous communities must be prioritized, which require clear acknowledgment of the presence of colonialism and the ongoing ramifications of colonial intervention in communities, infrastructure, institutions, and research, in order to mitigate centuries of oppression.

There is a need to decolonize research, implementation, and evaluation design in order to ensure that the work being done is sustainable and effective, and the data collected are ethical, reliable, and replicable [24]. There is a lack of information and research on the shared characteristics of decolonized methodologies and the tools and measures of outcomes and success. This review will not assess the “efficacy” of programming in comparison with other research methodologies but rather seek to identify shared (or differing) research and data collection methods, evaluation strategies, outcome measurement, and other indicators and outcomes, and how effectively they incorporate community voices. This is the first step in developing a shared understanding of best practice in decolonized sexual health research.

An initial scan of PROSPERO, MEDLINE, Open Science Frameworks, and Joanna Briggs Institute (JBI) Database of Systematic Reviews and Implementation Reports has been conducted, and there are no past or ongoing reviews on shared characteristics of decolonized research methodologies in the context of SRH. While there have been reviews of the characteristics of specific indigenous research methodologies [25] and models of service delivery [26], participatory action research for SRH [27], and meta-reviews of the different forms of community-engaged scholarship [9], there still remains a gap in the assessment of what it means to decolonize SRH research and what tools and assessments of success researchers are using.

### Objectives

There is no shared understanding of the characteristics that decolonized research methodologies share. We will address this gap in the literature by identifying the different characteristics of decolonized SRH research and interventions and the designs and methodologies on which they are based. The researchers will identify and map the tools used to define, measure, and analyze program outcomes and success in this context*.* Summarizing research strategies and findings will enable the researchers to identify not only recommendations for future research but also the space where there is room for growth in researching and designing decolonized SRH studies.

### Review Questions

Through this scoping review, the researchers will answer the following question: What are the characteristics of decolonized research methodologies in SRH research?

The primary outcome of this research will be to identify the shared characteristics of these methodologies, including a specific focus on the tools used to collect and analyze data. Secondary outcomes will include the specific measures and definitions of success, and how they are identified across geographic regions.

## Methods

This protocol was designed using the framework for scoping review from the JBI [28] and referred back to the reporting framework of PRISMA-ScR (Preferred Reporting Items for Systematic Reviews and Meta-Analyses Extension for Scoping Reviews) [29].

### Search Strategy

The search strategy will aim to locate peer-reviewed published studies (including ongoing studies) and gray literature on decolonized SRH research or program implementation and evaluation published between January 2012 and December 2022. The decision to use this time frame was based on the increasing recognition of various decolonizing methodologies over the last decade [9]. This decade of research will cover a great deal of relevant research conducted while ensuring an equal representation of research based on recent methodological ideologies and trends. There will be no geographic exclusion criteria, as decolonized research can be undertaken in any location. Abstracts, posters, book reviews, and blog posts will not be included.

A 3-step search strategy will be used to ensure that the strategy is comprehensive and collects as many appropriate studies as possible. The search strategy was developed in cooperation with a librarian scientist at McMaster University. First, a preliminary limited search of 2 web-based databases (eg, MEDLINE [Ovid] or JSTOR) will be conducted (Multimedia Appendix 1). Second, an analysis will be done of the text words in the title, abstract, and index to develop an updated search strategy. This search strategy using these terms will be adapted and applied to each relevant database. The databases searched include Embase, EMCare, MEDLINE (Ovid), Global Health Database, and Web of Science. Gray literature sources include Advanced Google, World Health Organization (WHO), United Nations Children’s Fund (UNICEF), United Nations Population Fund (UNFPA), Guttmacher Institute, and Médecins Sans Frontières (MSF). Finally, the resource lists of identified reports and papers will be reviewed for additional relevant studies.

### Study Selection

Following the search, all identified citations will be uploaded into DistillerSR: Literature Review Software (Evidence Partners), and duplicates will be removed. Titles and abstracts will then be reviewed by 2 or more independent reviewers for assessment against the inclusion criteria for review. Potentially relevant sources will be retrieved in full and uploaded to DistillerSR for subsequent review. The full text of the citations will be assessed against the inclusion criteria by the reviewers. Reasons for the exclusion of sources of evidence will be recorded and validated by another reviewer and reported in the full scoping review. If there are any disagreements during this process, they will be resolved through discussion or by involving additional reviewers. If any existing systematic or scoping reviews are identified during the screening, relevant studies will be extracted and added to the review if not already present. The results of the search and the study inclusion process will be reported in full in the final scoping review and presented in a PRISMA-ScR flow diagram to demonstrate the process of paper inclusion or exclusion.

### Data Extraction

The data extraction process for reporting will first take a quantitative approach, which will allow reviewers to identify key themes for exploration. These themes will subsequently be reported in narrative form. The reporting will be compliant with the PRISMA-ScR checklist.

The reviewers will use a data extraction tool developed by them for this scoping review. The data extracted will include bibliometric details, geographic location of primary institution, geographic location of research, study design, methodology, and community involvement, as well as key findings relevant to the study question. A draft data extraction form is provided in Multimedia Appendix 2. The draft data extraction tool will be modified and revised as necessary during piloting, and all changes will be detailed in the final scoping review. The piloting process will involve researchers gathering to discuss the data extraction form, testing individually on papers selected from the preliminary research search, and making adjustments for usability. Once approved, this will be entered into DistillerSR into at least 3 levels of analysis.

Any disagreements between reviewers will be resolved through discussion. If no consensus can be reached, a third reviewer will adjudicate. Data will also be subject to quality control checks. If necessary, authors of papers will be contacted to request missing or additional data.

### Analysis and Reporting

We will conduct quantitative analysis of the extracted data including descriptive statistics regarding areas of publication, institutions, income level and geographic location, and qualitative analysis of content and themes. The results will be presented in tables, charts, and figures, and a narrative summary of findings will represent the primary analysis and reporting.

Paper details will be presented in a table showing authors names, year research was published, location of research, aim or objective of study, methodology, and results. Charts and visualizations developed via descriptive statistics will be used to represent geographic location of lead author affiliations, location of funding institution, location of research, and comparison of these three. A narrative discussion of the results around shared characteristics between methodologies, data collection methods, study outcomes, community engagement strategies and leadership, development of data collection tools, and determinations of success will also be included. Thematic synthesis may be required to discuss these elements. If so, the reviewers will extract the key concepts from the text, develop descriptive codes for themes that carry across studies, and produce final analytical themes from this process [30]. The authors will further use these data to comment on unexpected findings, identified gaps, and implications for research and practice.

A narrative summary will accompany the charted results and will describe how the data relate to the stated objective and question, namely identifying shared characteristics of decolonized SRH findings. The summary will also discuss the ways in which the voices of the community of focus are centered and prioritized as well as other thematic areas that emerge over the course of the research. The team will work together to identify gaps in the research and present recommendations for future research and work in this area.

### Ethics and Dissemination

Ethics approval is not required for a scoping review as the materials being used are publicly available. Dissemination will be done through conference presentation, publication in a peer-reviewed open-access journal, and dissemination among researchers and policy makers in the field of SRH research and practice. Input from leaders and practitioners in this field will be requested to support dissemination. Findings will also be available as an element of a doctoral thesis.

## Results

The search strategy netted an initial 4967 studies for title or abstract review after the removal of duplicates. This initial review was conducted on Covidence and was completed in November 2022. In total, 1847 studies were marked for inclusion and exported to DistillerSR where a subsequent 70 duplicates were identified. The remaining 1777 studies were sent to a second round of title or abstract review, which was completed in January 2023. In total, 706 studies have been downloaded for full-text inclusion, which is expected to be completed by April 2023. We aim to complete data extraction and begin data analysis by May 2023. The final manuscript is expected to be submitted for publication by the end of July 2023.

## Discussion

### Principal Findings and Implications

The results from this scoping review will inform researchers and implementors as to the shared characteristics that decolonized methodologies in sexual health research share. In particular, in the field of SRH, where power and oppression play a critical role in the delivery and receipt of health care services, understanding how current interventions acknowledge and address this through their methodologies will provide guidance on future work. Findings from this study will provide guidance as to where there are gaps in defining and implementing decolonized research methodologies throughout the research process. Findings will also show where there are well-established methodologies for decolonizing traditional research practices, providing the first step toward the development of clear guidance for others hoping to replicate and expand on this style of research.

As the importance of decolonizing research evolves and expands, it will become important for a broad range of stakeholder groups to have access to a set of standards or recommendations regarding best practice. The findings from this scoping review will help to identify the commonalities that unite various forms of decolonized research and create a foundation for research strategies to be designed and used alongside communities of focus. Knowledge from this review may also inform researchers in the design and implementation of their own work as a point of reference for application of the strategies identified therein. Findings from this scoping review will support the existing theoretical literature from scholars such as Battiste [7] and Smith [5], who describe and identify the need for decolonized methodologies, and set the agenda for future work.The results of this analysis will support the findings of other literature which identifies the shared characteristics of related research methodologies [25] and identifies common strategies to guide researchers in the implementation of specific approaches [27], to create consensus. The findings from this review will add to the growing body of work describing and supporting the practical uses of new theories and methodologies [31].

### Limitations

The primary limitation of this work is the challenge of uncovering interventions and research that may not have been conducted within the guidelines of dominant culture publication and academic authorship. A significant limitation of the search strategy was that the structure of a scoping review and the academic language used may limit the inclusion of materials outside of academic institutions. While open-source databases and other web-based resources were accessed in an attempt to identify non–peer-reviewed materials, community-led and organized interventions are less likely to have been identified and included based on how data on these projects are published and shared.

### Conclusions

There remains a gap in the research on the meaning and application of decolonized research strategies, particularly within SRH. The findings of this study will contribute to a shared definition of decolonized methodologies and how they can be applied as a common practice in global health research. The study will inform the design and implementation of future decolonized research and evaluation strategies, particularly around SRH. Potential stakeholders who may use these findings include researchers, community-based organizations, and individuals within communities of focus.

## Appendix

Multimedia Appendix 1 Search strategy.

Multimedia Appendix 2 Data extraction framework.

## Abbreviations

JBI: Joanna Briggs Institute
MSF: Médecins Sans Frontières
PRISMA-ScR: Preferred Reporting Items for Systematic Reviews and Meta-Analyses Extension for Scoping Reviews
SRH: sexual and reproductive health
UNFPA: United Nations Population Fund
UNICEF: United Nations Children’s Fund
WHO: World Health Organization

## Data Availability

The data sets that will be generated and analyzed during the course of the study will be attached as a supplementary document to the final study or if the size of the data set makes this challenging will be available from the corresponding author on reasonable request.
