# Supplementary material for: Decolonization in Sexual and Reproductive Health Research Methods: Protocol for a Scoping Review
Source: JMIR Res Protoc. 2023 Apr 14;12:e45771. doi: 10.2196/45771 (PMC10148217; doi:10.2196/45771)
Supplement: Multimedia Appendix 1 [file resprot_v12i1e45771_app1.docx]

| **Concept** | **Alternate Search Terms** |
| --- | --- |
| Decolonized Methodologies | Decoloniz* research, indigenous research, community based participatory research, community based research, community-led research, empowerment research, participatory action research, culturally grounded, citizen science, culturally sensitive research, grounded theory, grassroots research, postcolonial, anti-racist, critical theory, feminist theory, emancipatory research |
| Sexual and Reproductive Health | family planning/services, sexual health, reproductive health, prenatal, neonatal, antenatal, pregnancy, birth, Sexually Transmitted Infections (STIs), sexually transmitted diseases (STDs), birth, gender based violence, intimate partner violence, abortion, sexual behavior, sexual and gender minorities, sexual identity, menstrual health, abortion, HIV/AIDS, contraception/ives, HPV, female genital mutilation, circumcision, sexual education, reproductive health, herpes, chlamydia, syphilis, gonorrhea, maternal health services, clitoral cutting, fertility, pregnancy complications, cervical cancer, sex education, contraceptive devices, birth control, rod implant, sexual wellness, child marriage, sexual behavior, infertility |
| Decolonized Methods (forms of data collection) | Photovoice, participatory visual methods, community mapping, ethnography, interviews, body mapping, surveys, questionnaires, surveillance, focus groups, narrative, storytelling, phenomenology, observation, case study, oral tradition, qualitative |
