# Supplementary material for: Decolonization in Sexual and Reproductive Health Research Methods: Protocol for a Scoping Review
Source: JMIR Res Protoc. 2023 Apr 14;12:e45771. doi: 10.2196/45771 (PMC10148217; doi:10.2196/45771)
Supplement: Multimedia Appendix 2 [file resprot_v12i1e45771_app2.docx]

| **Main Category** | **Sub Category** | **Description** |
| --- | --- | --- |
| Author (s) |  |  |
| Lead Author |  | Indicate institution where author is based, country of primary affiliation |
| Funders |  | Indicate funder country of origin |
| Year of Publication |  | The year the research was published |
| Country where study was conducted |  | Indicate country/countries where research took place, and income level based on World Bank classification |
| Aims/Objectives of study |  | Describe stated aim of study |
| Methodology | Study Design: Sub-category of Quantitative, qualitative, or mixed methods | Specify study design of research within stated category (e.g. cross-sectional, ethnographic, exploratory, etc.) |
|  | Population | Age range, sex, inclusion criteria to participate in study (e.g. pregnancy, HIV+) |
|  | Data Collection Method | Specify methods of data collection |
|  | Data Collection/Evaluation tools | Specify types of tools used if applicable, and if developed/adapted specifically for this research |
|  | Outcome Measures | Indicate primary and secondary outcomes (where applicable), or selected measures of success (e.g. how were objectives of study measured) |
|  | Community Involvement | Was the community involved in development of methods or data collection tools (e.g. key stakeholder involvement in design), co-designing activities |
|  | Data Analysis | Method of data analysis |
| Results | Reported Outcomes | Key SRH outcomes OR quantitative or qualitative results related to primary stated outcome |
|  | Key Findings | Summarize any key findings that inform decolonization methodologies and their efficacy |
